# High-Throughput Sequencing Uncovers Fungal Community Succession During *Morchella sextelata* Development

**DOI:** 10.3390/jof11050364

**Published:** 2025-05-07

**Authors:** Qi Yan, Peng Wang, Zhushan Liu, Ya Yu, Xiao Tan, Xiao Huang, Jiawei Wen, Weidong Zhang

**Affiliations:** 1 College of Horticulture, Jilin Agricultural University, Changchun 130118, China; 20231362@mails.jlau.edu.cn; 2 Jilin Academy of Agricultural Sciences (Northeast Agricultural Research Center of China), Changchun 130033, China; wangpeng@cjaas.com (P.W.); liuzhushan@cjaas.com (Z.L.); yuya@cjaas.com (Y.Y.); tanxiao@cjaas.com (X.T.); huangxiao@cjaas.com (X.H.)

**Keywords:** *Morchella sextelata*, diversity, soil fungal community, high-throughput sequencing

## Abstract

To investigate the correlation between soil fungal communities and the growth and development of *Morchella sextelata*, this study utilized high-throughput sequencing technology to analyze the structure and diversity of soil fungal communities at various growth stages of *Morchella sextelata*. The results revealed significant variations in the diversity, composition, and relative abundance of soil fungal communities across different growth stages of *Morchella sextelata*, demonstrating stage-specific characteristics. Alpha diversity analysis indicated that the Shannon index was highest during the CK stage, significantly decreased in the LS stage (*p* < 0.01), increased again in the LY stage, and then declined once more in the LC stage. Beta diversity analysis (Principal Coordinates Analysis, PCoA) demonstrated significant differences in fungal community structure across various stages (R = 0.9567, *p* = 0.001). At the phylum level, *Ascomycota* remained dominant throughout all growth stages of *Morchella sextelata*, but its relative abundance exhibited significant dynamic changes. At the fungal genus level, *Paecilomyces* dominated in the primordium stage (27.12%), whereas *Morchella* dominated in the conidial stage (LS) and fruiting body stage (LC), accounting for 43.48% and 41.61%, respectively. Additionally, in the LC stage, the plant pathogenic genus *Fusarium* significantly increased (3.49%), indicating an elevated risk of disease. Functional prediction results revealed that saprotrophic fungi were predominant at all stages, but the relative abundance of pathogenic fungi gradually increased, rising from 0.06% in the LS stage to 41.41% in the LC stage, a substantial increase of 40.81% compared to the LS stage. This suggests a higher potential risk of disease occurrence during the fruiting body stage. Our study provides an overview of the dynamics of soil fungal communities during the cultivation of *Morchella sextelata*. These findings offer scientific insights for optimizing the artificial cultivation technology of *Morchella sextelata* and provide a reference for disease prevention and control.

## 1. Introduction

Morels (*Morchella* spp.) are highly prized edible and medicinal fungi known for their unique honeycomb-like structure and rich content of amino acids, polysaccharides, and trace minerals [1,2,3,4]. As a globally renowned rare edible and medicinal fungi, morels have high nutritional and cultivation value. At present, the artificial cultivation technology of morels is not yet fully mature, which is mainly manifested in unstable yield, long growth cycles, and difficulties in the prevention and control of pests and diseases [5]. Recent studies have shown that *Agaricus bisporus* [6,7], *Ganoderma lucidum* [8,9], *Phlebopus portentosus* [10], and *Stropharia rugosoannulata* [11], mushrooms that depend on the soil cover for their growth and development, are strongly influenced by the soil microbial community. These microorganisms play a wide range of important roles, including effective prevention of pathogen infestation, facilitation of transport and cycling of substances in the soil to support mushroom growth, and induction of the formation of basidiomycetes [12,13,14].

The soil microbial community plays a crucial role in the growth and development of *Morchella sextelata*. In contrast to most edible fungi, which are cultivated in pre-sterilized substrate packages, *Morchella sextelata* is grown directly in soil beds. Consequently, its mycelial growth and fruiting body formation are significantly influenced by the soil microbial community [15]. Previous studies have shown a positive correlation between the formation of fruiting bodies and the diversity and evenness of soil microbial communities during large-scale cultivation of *Morchella sextelata* [16]. Sustained propagation of *Morchella sextelata* acidifies soils, enhances the richness and diversity of fungal and bacterial assemblages [17]. In soils with low or no *Morchella sextelata* yield, some pathogenic fungi accounted for a high proportion, including *Gibberella*, *Microidium*, *Penicillium*, *Sarocladium*, *Streptomyces*, and *Trichoderma* [18].

The structure and function of soil microbial communities show a high degree of complexity and dynamic characteristics, with both advantages and disadvantages. Certain beneficial microorganisms can effectively enhance the yield and quality of morel mushrooms, while pathogenic microorganisms or competitive fungi may inhibit their growth. Therefore, the present study analyzed the soil fungal diversity and community structure of *Morchella sextelata* at different growth stages using ITS high-throughput sequencing technology, in order to provide a reference for the optimization of artificial cultivation techniques and pest control. We hypothesized that the growth and development of *Morchella sextelata* would significantly alter the diversity, composition, and function of soil fungal communities across different growth stages. Specifically, we expected beneficial fungi to dominate in the early stages and pathogenic fungi to increase in later stages. Our results confirmed this hypothesis, revealing that the diversity of fungal communities was highest in the bare soil stage and declined after *Morchella sextelata* planting. The relative abundance of *Ascomycota* showed dynamic changes across stages, with *Paecilomyces* dominating in the primordium stage, while *Morchella* was most abundant in the later stages. Furthermore, the relative abundance of the pathogenic genus *Fusarium* increased significantly in the fruiting body stage. These findings offer novel insights into soil microbial dynamics during *Morchella sextelata* cultivation.

## 2. Materials and Methods

### 2.1. Experiment Description and Sampling

**Figure 1:**
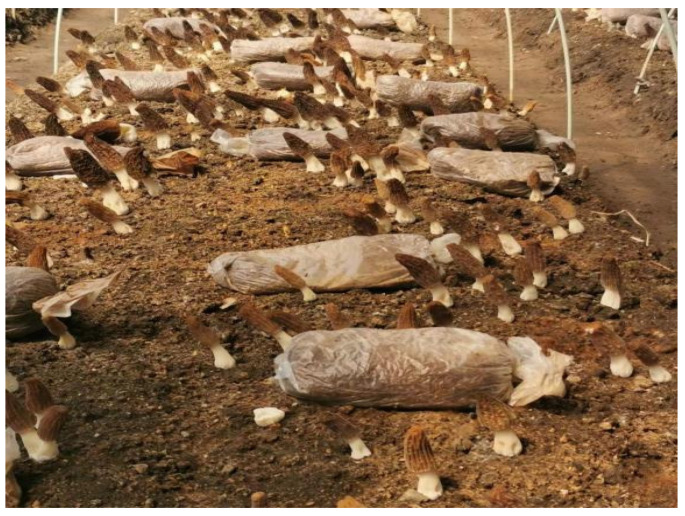
Morphology and cultivation environment of *Morchella sextelata* fruiting body.

In November 2023, the cultivation practice of *Morchella sextelata* was carried out in Dongfeng County, Liaoyuan City, Jilin Province (Figure 1). Prior to planting, a layer of quicklime was evenly applied to the greenhouse soil surface at a rate of 3750 kg/ha [5]. Soil preparation involved the method of transverse ridge making, with ridges 7 m long, 1 m wide, and 20 cm spacing between them.

During the entire cultivation process, environmental parameters including soil temperature (maintained at 6–18 °C during early stages and 8–12 °C during later stages), air humidity (kept at 85–90%), and light intensity (controlled by 60–70% shading net) were carefully regulated to ensure stable growing conditions.

The ground temperature in the greenhouse was kept within the appropriate range of 6–18 °C for artificial sowing of morel strains, which had been evenly spread on the leveled surface of the ridge and covered with soil. On the 10th day, exogenous nutrient bags were placed on the soil surface and subsequently covered with black plastic film. On day 40, white conidia emerged from the soil surface, and soil samples were collected (referred to as conidial stage samples (LS)) [19]. On the 59th day, the induction stage for the fruiting bodies of *Morchella sextelata* was then initiated. The soil temperature was maintained between 8 and 12 °C, while the relative humidity of the air was kept at 85–90%. A sunshade net was installed on the side of the greenhouse, with a shading rate controlled at 60–70%, to facilitate the formation of *Morchella sextelata* primordium [20]. On the 69th day, spherical primordia with a diameter of 0.5 mm–1 mm appeared on the ridge surface, and soil samples were collected (referred to as primordium stage samples (LY)). On the 84th day, when the fruiting body grows to more than 10 cm, the cap diameter reaches more than 2 cm, and the color changes from dark gray to light gray or brownish yellow, the soil samples were collected (referred to as fruiting bodies stages samples (LC)).

**Figure 2:**
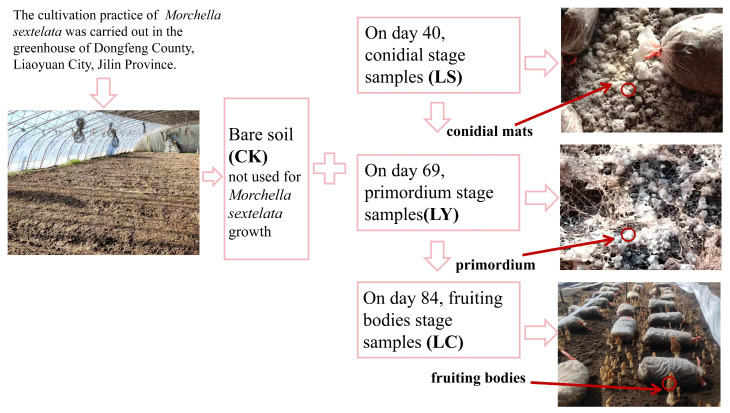
*Morchella sextelata* has different growth stages. CK: bare soil stage; LS: conidial stage; LY: primordium stage; LC: fruiting bodies stage.

A total of 12 soil samples were collected (Figure 2). The five-point sampling method was employed. Firstly, the midpoint of the diagonal line was identified as the central sampling point. Subsequently, four additional points, equidistant from the central point along the diagonal, were selected as the other sampling points [21]. A soil sampler with a diameter of 2.5 cm was utilized to dig approximately 10 cm deep at each sampling point. Topsoil was removed, and soil samples were collected at the bare soil stage, conidial stage, primordium stage, and fruiting bodies stage, respectively. At each stage, five soil samples (each to a depth of 10 cm) were collected and mixed to form a single composite sample. Three biological replicates were established at each stage, with bare soil CK (i.e., soil not utilized for *Morchella sextelata* cultivation) serving as the control group. The collected soil samples were placed in insulated ice boxes and promptly transported to the laboratory for storage at −80 °C until further use.

Although the physicochemical properties of the soil (such as pH, nutrient content, and organic matter) were not directly measured in this study, all soil samples were collected from a uniform, continuously managed greenhouse site to minimize environmental variation.

### 2.2. Soil DNA Extraction, PCR Amplification, and Sequencing

The CTAB (Cetyltrimethylammonium Bromide) method was employed to isolate total genomic DNA from the samples. For the amplification of the ITS internal transcribed spacer (ITS1-1F) region of fungi, the primers ITS1-1F-F (5′-CTTGGTCATTTAGAGGAAGTAA-3′) and ITS1-1F-R (5′-GCTGCGTTCTTCATCGATGC-3′) were used. Each PCR reaction mixture consisted of 15 µL of Phusion^®^ High-Fidelity PCR Master Mix (New England Biolabs, Inc., 240 County Road, Ipswich, MA, USA), 0.2 µM of each primer, and 10 ng of template DNA. The thermal cycling conditions included an initial denaturation at 98 °C for 1 min, followed by 30 cycles of denaturation at 98 °C for 10 s, annealing at 50 °C for 30 s, and exte, nsion at 72 °C for 30 s, with a final extension at 72 °C for 5 min.

The DNA amplification products were detected using Agilent 5400. The PCR products were then combined in equal proportions and purified using the Qiagen Gel Extraction Kit (Qiagen Gel Extraction Kit (Qiagen, Hilden, Germany). Sequencing libraries were prepared using the NEBNext^®^ Ultra™ II DNA Library Prep Kit (New England Biolabs, Inc., 240 County Road, Ipswich, MA, USA, Catalog #: E7645B). The quality of the libraries was evaluated using the Qubit^®^ 2.0 Fluorometer (ThermoFisher Scientific, Waltham, MA, USA) and the Agilent Bioanalyzer 2100 system (Agilent Technologies Inc., Santa Clara, CA, USA). Subsequently, the library was sequenced on an Illumina NovaSeq platform (Illumina Novaseq6000, Illumina, San Diego, CA, USA), generating 250 bp paired-end reads. The paired-end reads were assigned to their respective samples based on unique barcodes and trimmed to remove barcode and primer sequences. The FLASH software (Version 1.2.11, http://ccb.jhu.edu/software/FLASH, accessed on 18 July 2022) [22] was employed to merge the paired-end reads, which is a rapid and accurate tool designed to combine overlapping reads generated from opposite ends of the same DNA fragment. The merged sequences were designated as Raw Tags. Quality filtering of the raw tags was conducted using the fast software (Version 0.20.0) to obtain high-quality Clean Tags. The Clean Tags were compared with the reference database (Unite database 8.2, https://unite.ut.ee/ for ITS, accessed on 2 April 2020) using Vsearch (Version 2.15.0) to detect the chimera sequences, and then the chimera sequences were removed to obtain the Effective Tags [23].

### 2.3. Statistical Analysis

For the previously acquired Effective Tags, denoising was conducted using the DADA2 module within the QIIME2 [24] software package (version QIIME2-2020.06), with DADA2 serving as the default method to generate initial Amplicon Sequence Variants (ASVs). Subsequently, ASVs with an abundance below 5 were excluded from further analysis. Species annotation was facilitated by the QIIME2 software, utilizing the Unite Database specifically for Internal Transcribed Spacer (ITS) sequences.

Normalization of ASV absolute abundance was achieved by adopting a standard based on the sequence count of the sample with the fewest sequences. The sequence count after normalization is cutoff = 66,636. All subsequent analyses of alpha and beta diversity were based on this normalized dataset. To examine the diversity, richness, and evenness of microbial communities within the samples, alpha diversity indices were computed using four metrics available in QIIME2, including Chao1, Shannon, Simpson, and Good’s coverage [25]. Principal Coordinate Analysis (PCoA) was conducted to assess between-group community differences [26]. Prior to intergroup comparisons, the normality of alpha diversity indices was tested using Shapiro–Wilk test. Depending on data distribution, either parametric (T-test) or non-parametric (Wilcoxon rank-sum) tests were applied, with *p*-values corrected by FDR method when necessary. Beta diversity differences were assessed using PERMANOVA (adonis) and ANOSIM, both performed within QIIME2. Differentially abundant taxa at phylum and genus levels were identified using (MetaStat 1.3) and *t*-test analysis in R (version 3.5.3) [27].

Additionally, to explore the ecological functions of fungal communities and identify functional differences among groups, the literature-curated database of fungal ecological functions, (FunGuild v1.0), was employed. It was used to annotate amplicon-derived taxa and classify them into ecological guilds.

## 3. Results

### 3.1. Analysis of Sequencing Data of Soil Samples

**Figure 3:**
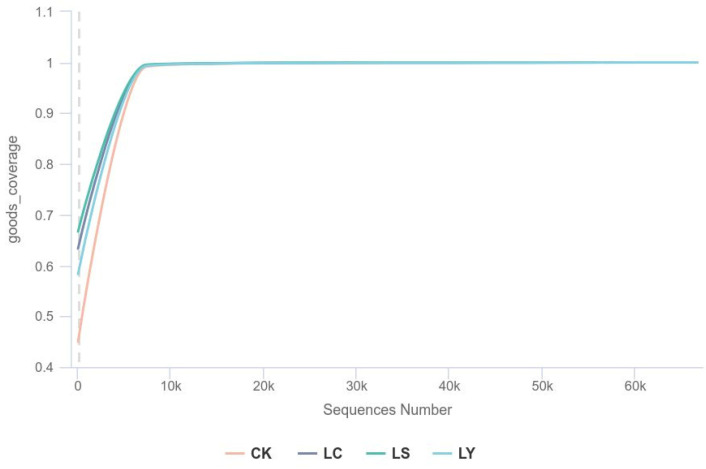
Sample rarefaction curves of soil fungal communities at different growth stages of *Morchella sextelata*. The dilution curve of each sample was drawn to reflect the rationality of sequencing data amount. When the curve tended to be flat, the amount of sequencing data was gradually reasonable, proving that the amount of sequencing data was saturated. CK: bare soil stage; LS: conidial stage; LY: primordium stage; LC: fruiting bodies stage.

After sequencing, a total of 911,551 optimized sequences were obtained from all samples. Based on the results of data processing, the reads were clustered using the DADA2 method [28] with 100% sequence identity to obtain ASVs (Amplicon Sequence Variants). Rarefaction curves for each sample were plotted, and these curves flattened out based on the minimum number of sample sequences. As depicted in Figure 3, the rarefaction curves of all samples transitioned to a flattened state after a sharp increase, and the library coverage exceeded 0.99, suggesting that the sequencing depth for all samples was adequate.

### 3.2. Changes in Fungal Community Diversity

**Figure 4:**
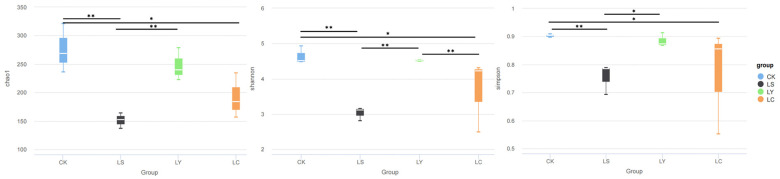
The diversity of soil fungal communities during different growth stages of *Morchella sextelata*. The Chao1 index represents species richness, the Shannon index represents uniformity, and the Simpson index represents diversity. CK: bare soil stage; LS: conidial stage; LY: primordium stage; LC: fruiting bodies stage. Statistical significance was determined using the Wilcoxon rank-sum test with FDR correction. * *p* < 0.05, ** *p* < 0.01.

Alpha diversity analysis was conducted to assess the diversity and richness of soil fungal communities associated with *Morchella sextelata* during its growth period. Across all planting stages, the α diversity of soil fungal communities decreased in the two developmental stages called LS and LC, whereas it increased sharply in the LY stage (Figure 4). Notably, the fungal diversity in CK soil was the highest and significantly differed from that in LS soil (*p* < 0.01). Furthermore, after the planting of *Morchella sextelata*, the soil fungal diversity decreased sharply (Figure 4). These results demonstrate that the diversity and abundance of soil fungi undergo dynamic changes as *Morchella sextelata* grows and develops, initially increasing and then decreasing.

Alpha diversity analysis was performed to evaluate the richness and diversity of soil fungal communities at different growth stages of *Morchella sextelata* cultivation (Figure 4). The Chao1, Shannon, and Simpson indices revealed significant variation in fungal community diversity across the four sampling groups: CK (bare soil stage), LS (conidial stage), LY (primordium stage), and LC (fruiting body stage). Overall, the CK group exhibited the highest fungal diversity, as indicated by all three indices. Compared to CK, the LS group showed a significant decrease in Chao1 (*p* < 0.01), Shannon (*p* < 0.01), and Simpson indices (*p* < 0.01), suggesting a sharp decline in fungal richness and diversity following the introduction of *Morchella sextelata*. During the LY stage, all three indices increased significantly compared with LS (Chao1: *p* < 0.01; Shannon: *p* < 0.01; Simpson: *p* < 0.01), indicating a temporary recovery in fungal diversity during primordium formation. However, a subsequent decline was observed at the LC stage, where Chao1 and Shannon indices were significantly lower than those in LY (*p* < 0.05). These findings suggest that the cultivation of *Morchella sextelata* leads to dynamic and stage-specific changes in the soil fungal community, characterized by an initial decline in diversity after inoculation, a transient increase during primordium formation, and a final decline at the fruiting body stage.

**Figure 5:**
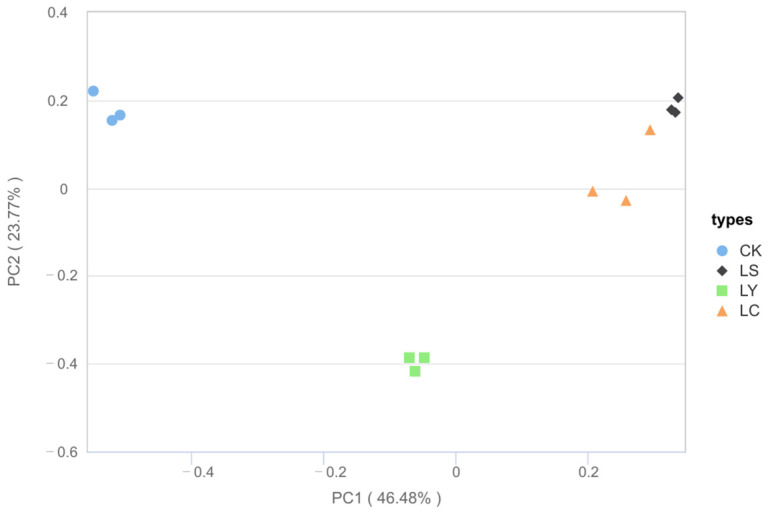
PCoA sorts the eigenvalues based on the distance matrix. Different colors are used to represent different samples. CK: bare soil stage; LS: conidial stage; LY: primordium stage; LC: fruiting bodies stage. R = 0.9567, *p* = 0.001000.

Principal Coordinates Analysis (PCoA) is a method utilized for beta diversity analysis to examine the similarities and differences in the composition of sample communities. The closer the sample distance, the more similar the microbial composition and structure between the samples, and the smaller the difference. The closer the sample distance, the more similar the composition and structure of the species. Therefore, samples with high community structure similarity tend to gather together, and samples with large community differences tend to be farther apart. Significant variations were observed in the composition of soil fungal communities across different growth stages of *Morchella sextelata* (R = 0.9567, *p* = 0.001). As illustrated in Figure 5, the percentages of variance explained by the first and second principal coordinate axes were 46.48% and 23.77%, respectively, totaling 70.25%. On the PCoA plot, the CK group did not overlap with the LS, LY, and LC groups, indicating a significant divergence in fungal microbial community structure between uncultivated soil and soil after *Morchella sextelata* cultivation. In the PCoA diagram, the soil fungal communities with shorter distances between LS and LC exhibited the highest similarity, whereas those with greater distances between LY and CK, as well as between LS and other groups, demonstrated significant differences. This suggests that the fungal communities in the LS and LC stages exhibited aggregation, whereas the structure of the fungal communities in the LY stage differed markedly. These results reveal that during the different growth stages of *Morchella sextelata*, the composition and structure of soil microbial communities undergo substantial changes.

### 3.3. Changes in Fungal Community Composition

**Figure 6:**
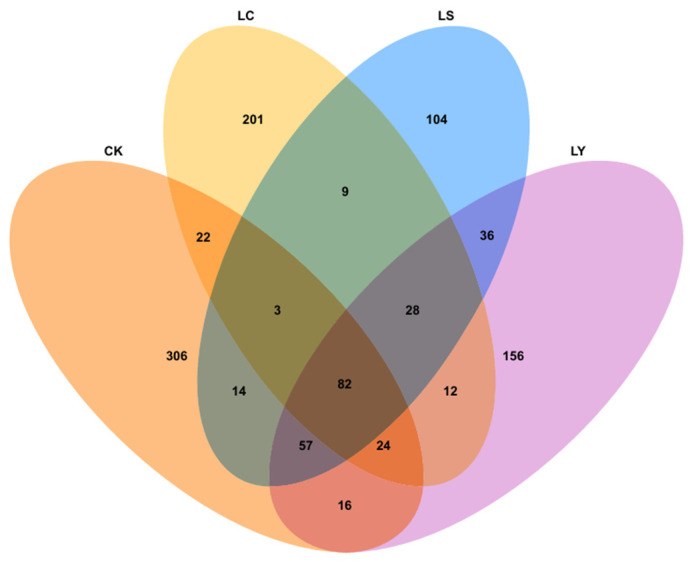
Each circle in the figure represents a sample group. The numbers in the overlapping parts of the circles represent the number of ASVs shared between the sample groups, and the numbers in the non-overlapping parts represent the number of ASVs unique to the sample groups. CK: bare soil stage; LS: conidial stage; LY: primordium stage; LC: fruiting bodies stage.

The Venn diagram can intuitively reflect the specificity and overlap of species composition among different growth periods. The classify-sklearn algorithm in QIIME2 was used to classify the representative sequences of ASVs (Amplicon Sequence Variants) with a 100% similarity threshold. The number of fungal ASVs in the CK, LC, LS, and LY groups was 524, 318, 333, and 411, respectively (Figure 6). There were 82 ASVs shared among the four groups of soil sample fungi. Specifically, the highest number of unique fungal ASVs was 306 in the CK group. During the growth stages of *Morchella sextelata*, the highest number of unique fungal ASVs in the LC group was 201, followed by 156 unique fungal ASVs in the LY group, and the lowest number was 104 unique fungal ASVs in the LS group. The research results indicate that a portion of fungal species are ubiquitous across different growth stages and in soil without morel cultivation, potentially forming the core component of the soil fungal community. Furthermore, as the growth stages of *Morchella sextelata* change, the number of unique fungal ASVs in each group also varies, further proving that the impact of *Morchella sextelata* cultivation on soil fungal species composition is dynamically changing.

**Figure 7:**
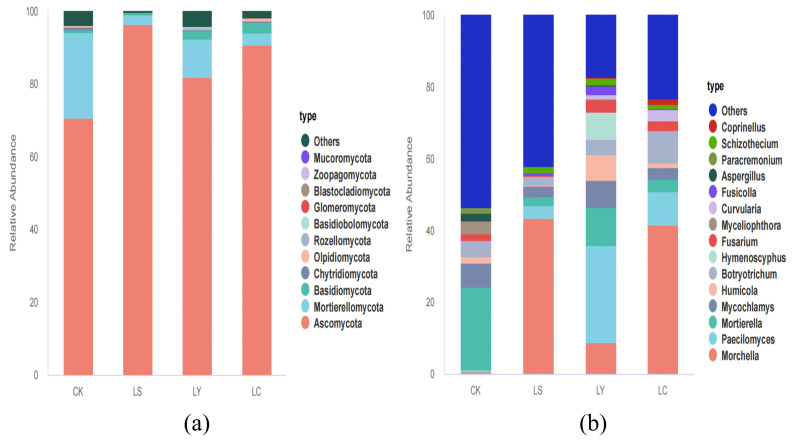
Stacked bar plots illustrating the composition of fungal colonies in soil at the phylum level (**a**) and at the genus level (**b**). CK: bare soil stage; LS: conidial stage; LY: primordium stage; LC: fruiting bodies stage.

At the phylum level, all species are considered for classification; at the genus level, only the top 15 most abundant taxa are considered for classification, with the remaining taxa classified as “others”. Figure 7a depicts eleven dominant fungal taxa annotated at the phylum level. Across all four sample types, *Ascomycota* was the dominant phylum, followed by *Mortierellomycota*. After planting *Morchella sextelata* soil, *Ascomycota* abundance gradually increased, while *Mortierellomycota* abundance gradually decreased. During each growth stage of *Morchella sextelata*, *Ascomycota* remained the dominant phylum, but its relative abundance exhibited significant dynamic changes. From the LS to LY growth and development stages, the abundance proportion of *Ascomycota* gradually decreased, with a slight rebound during the LC stages. As shown in Figure 7b, in the horizontal control (CK) group, the dominant genus was *Mortierella*, with a relative abundance of 23.03%. However, after planting *Morchella sextelata*, the abundance of *Mortierella* significantly decreased to 0.02%. Observing the different growth stages of *Morchella sextelata*, we found that during the LS and LC stages, the dominant genus shifted to *Morchella*, with relative abundances of 43.48% and 41.61%, respectively. In contrast, during the LY stages, the dominant genus changed to *Paecilomyces*, with its relative abundance significantly increasing to 27.12%. Further analysis of fungal taxon abundance changes revealed that the changes during the LY and LC stages were the most pronounced. The abundance of *Hymenoscyphus*, *Humicola*, *Fusarium*, and *Coprinellus* significantly increased during the LY stages. Meanwhile, during the LC stages, the relative abundances of *Botryotrichum*, *Coprinellus*, and *Fusarium* also showed significant increases.

### 3.4. Functional Prediction of Microorganisms

**Figure 8:**
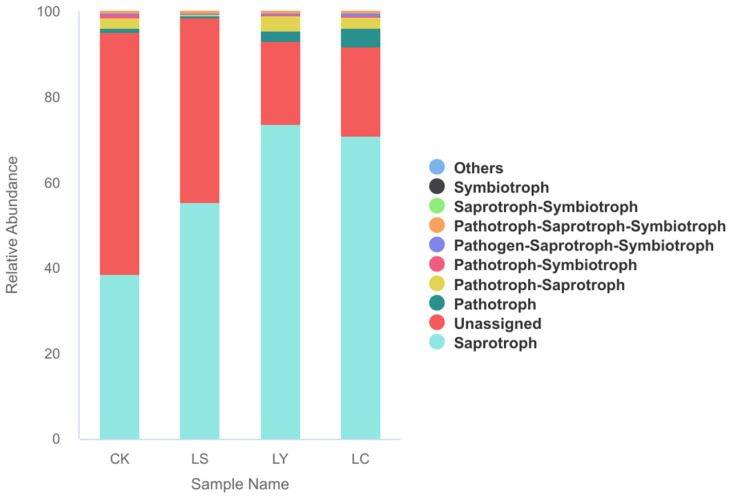
Stacked bar plots illustrating the functional prediction of soil fungi. CK: bare soil stage; LS: conidial stage; LY: primordium stage; LC: fruiting bodies stage.

The FUNGuild platform was utilized to predict and analyze the fungal functional groups (Figure 8). Based on their nutritional types, these functional groups were categorized into seven distinct classes: pathotroph–saprotroph–symbiotroph, saprotroph, saprotroph–symbiotroph, pathotroph–saprotroph, pathotroph, pathotroph–symbiotroph, and symbiotroph. Excluding unassigned categories, saprotrophic fungal metabolism was the predominant function, indicating the abundant presence of saprotrophic fungi. Their primary role is to facilitate the formation and decomposition of soil humus, thereby promoting carbon and nitrogen cycles. The nutritional types of *Morchella sextelata* exhibited varying trends across different growth stages. The prediction results indicate that the relative abundance of pathotrophs gradually increased, reaching a peak of 41.41% in the LC stage. This represents a significant increase of 40.81% compared to the LS stage.

## 4. Discussion

Under current cultivation techniques, mulching the soil is crucial for the development of morels. The microbial community within the soil varies with the fertility cycle of *Morchella sextelata*, resulting in alterations to the soil habitat [29]. Soil microbial activity and diversity significantly impact the growth and development of *Morchella sextelata*. Soil microbes influence plant diversity through competition, coordination, and by driving nutrient cycling [30]. Certain fungi may develop into pathogenic organisms that can cause diseases in host plants [31,32]. The presence of pathogenic fungal communities has been demonstrated in studies on Agaricus diseases, for example, *Fusarium* causes stalk rot, and *Paecilomyces penicillatus* is considered the causal agent of white mold [32,33]. This underscores the particular importance of fungal communities for cultivating *Morchella sextelata*. Therefore, exploring the changes in soil fungal communities across different growth stages of *Morchella sextelata* and understanding their interactions will aid in better utilizing the positive indirect effects of soil fungal communities on the cultivation of *Morchella sextelata*.

High-throughput sequencing results showed that the diversity, composition, and relative abundance of soil fungal communities of different growth stages of morel mushrooms varied significantly and were stage-specific. From the results of the α diversity index, it can be seen that the abundance and diversity of fungal microorganisms reached the highest level in the bare soil control (CK) samples, while the diversity of the fungal community was significantly reduced after planting *Morchella sextelata*. This is consistent with the results of YU et al. [18] and Tan et al [16]. It indicates that the growth of *Morchella sextelata* can seize the ecological status of the original dominant fungal communities in the soil, which prompts some of the original fungal communities to be suppressed. During the primordium stage (LY) of morel mushrooms, soil fungal diversity peaks throughout the entire growth cycle, whereas fungal diversity declines during the fruiting body stage (LC). This observation is consistent with the research findings of Zhang et al. [15]. This may be related to the protocorm stages’ special metabolic activities and nutritional requirements. As a key stage in the growth of *Morchella sextelata*, the protobasal stage may be through the release of specific nutrients and active components. However, as the substrates mature and these substances are depleted, soil fungi’s colonization may be somewhat inhibited. Similar patterns of microbial fluctuation during different developmental phases of morels have been observed in studies by Liu et al. [5], suggesting that this is a common feature in morel cultivation under diverse environmental and substrate conditions. Fan et al. [34] also indicated a positive correlation between the diversity of microorganisms involved in the ecological cycle in the soil and crop yield. However, after the cultivation of *Morchella sextelata*, microbial diversity showed a decreasing trend despite the increase in the relative abundance of fungal communities in the soil, which may be a potential reason for the decrease in yield when morel mushrooms are cropped in a continuous crop. Beta diversity analyses further revealed the differences in the soil fungal communities at different growth stages of *Morchella sextelata*. The results showed that the soil fungal communities showed high similarity and aggregation during the LS and LC stages, which may be related to the gradual accumulation of nutrients and ecological niche differentiation during the growth of the *Morchella sextelata* (Figure 5). These beta diversity patterns were also reflected in the studies of Zhang et al. [15], who noted niche convergence and microbial clustering during the fruiting stages of morel cultivation. Future studies need to further explore the specific mechanisms and ecological impacts behind these changes.

Analysis of the structure and composition of soil fungal communities at different growth stages of *Morchella sextelata* reveals a certain degree of specificity and overlap in species composition among these stages. At the phylum level, the dominant fungi in all soil samples belong to *Ascomycota*, followed by *Mortierellomycota*. From the LS to the LY stages, the abundance of *Ascomycota* gradually decreases and then gradually increases again by the LC stages. This suggests that *Ascomycota* constitutes the core successional fungal community, with its abundance dominating other communities and persisting throughout the LS, LY, and LC stages of *Morchella sextelata*. This result is not surprising, as *Ascomycota* dominates soil fungal communities globally. In the context of soil ecology, the dominance of *Ascomycota* in soil fungal communities is well-documented, as highlighted by Eleonora et al. [35], who emphasized the role of *Ascomycota* in various ecological functions, including nutrient cycling and decomposition. Previous studies have shown that *Ascomycota* is crucial in decomposing lignin and keratin in soil, promoting the transformation and cycling of soil nutrients and improving soil quality [36,37]. The microbial contribution to soil C storage is directly related to microbial community dynamics and the balance between formation and degradation of microbial byproducts [38]. The succession patterns observed in this study align with the general principles of soil microbial community assembly, where certain taxa dominate at different stages due to their specific ecological roles and environmental adaptations [38,39]. At the genus level, *Morchella* was the dominant genus during the LS and LC stages throughout the entire growth cycle of *Morchella sextelata*, while *Paecilomyces* was the dominant genus during the LY stages. The most significant changes in fungal community abundance occurred between the LY and LC stages. During the LY stage, there were notable increases in the abundance of *Hymenoscyphus*, *Humicola*, *Fusarium*, and *Coprinellus*. Meanwhile, during the LC stages, there was a trend of significantly increased relative abundance for *Botryotrichum*, *Coprinellus*, and *Fusarium*. This indicates the succession of soil fungal communities during the cultivation of *Morchella sextelata*. Furthermore, the community composition at the genus level revealed that soil fungal communities tended to be more diverse and complex during the LY and LC stages. The increase in the abundance of *Fusarium* during the LY stage, despite its potential pathogenicity, underscores the complexity of soil microbial interactions. According to Tedersoo et al. [39], such shifts in fungal community composition can have significant implications for plant health and ecosystem functioning.

Studies have shown that *Fusarium*, as a plant pathogen, infects various plants and causes symptoms ranging from poor growth and development, fruit or seed rot, chlorosis and wilting of leaves, and ulcers to root or stem rot, potentially triggering soil-borne diseases [40]. Additionally, some microorganisms in this study remained unidentified, a phenomenon commonly observed in previous research reports, suggesting that there are still a large number of unknown fungal taxa present in morel mushroom soil.

During the primordium formation stages, insufficient nutrients required for the growth of *Morchella sextelata* in the soil led to a change in dominant genera, and the placement of exogenous nutrient bags allowed *Morchella* to regain its dominant position in the soil during the LC stages, which is consistent with the research by LIU et al. [5]. Studies have shown that during the primordium formation of *Morchella sextelata*, bacteria from multiple phyla, such as *Proteobacteria*, *Actinobacteria*, and *Acidobacteria,* are promoted. These bacteria can inhibit the growth of soil pathogens and degrade toxic and harmful substances in the environment. Therefore, increased soil fungal diversity during primordium formation indirectly supports this view [15,41]. This study found that during the LY of *Morchella sextelata*, the relative abundance of the *Paecilomyces* genus significantly increased in the soil. This change may indicate a nutritional competition, symbiotic relationship, or beneficial microbial activities between this genus and the growth and development of *Morchella sextelata*. Notably, when the growth of *Paecilomyces* coincides with an increase in the proportion of other pathogen-related fungal genera (such as *Acremonium* and *Mortierella*), the disease risk may intensify further [42]. Additionally, studies have shown that the pathogen load of morels may increase from the primordium stages to the fruiting body production stages, with higher proportions of certain fungal genera (such as *Acremonium*, *Mortierella*, and *Paecilomyces*) in most unfruitful soils [15,16]. Research by HE et al. [43] indicates that white mold disease in morels is caused by *Paecilomyces penicillatus*. Furthermore, studies by YU et al. [44] further confirm that the presence of *Paecilomyces penicillatus* in the soil affects the occurrence of white mold disease in morels and significantly impacts their fruiting yield. These research results suggest that the balance of soil microbial communities is crucial for maintaining healthy growth and enhancing the yield of *Morchella sextelat*. Any microbial activities that disrupt this balance may adversely affect the growth cycle and final yield of *Morchella sextelat.* Therefore, regulating the soil microbial community structure and inhibiting the excessive growth of harmful microorganisms are important strategies to ensure high and stable yields of *Morchella sextelat* during artificial cultivation.

Using FUNGuild to predict the functions of soil fungi during different growth stages of *Morchella sextelat*, the results indicated that saprotrophic fungi dominate, while the relative abundance of pathotrophic fungi gradually increases, reaching as high as 41.41% during the LC stages. Saprotrophic fungi are prone to carrying pathogens, and the high abundance of pathotrophic fungi may imply a higher risk of disease for morel mushrooms during the fruiting body stages. This suggests an increased potential for soil-borne pests and diseases. Therefore, in field management, attention should be paid to preventing soil-borne disease infection, and appropriate types of soil conditioners should be selected. These predictions align with prior studies such as those by Liu et al. [5], who also observed functional shifts linked to disease risks during the fruiting stages. Further exploration will be conducted in future studies. This study was conducted within a single cultivation cycle and region, potentially limiting the generalizability of the findings to broader ecological or geographical contexts. Additionally, microbial functional predictions were based on ITS sequence data and database annotations, which may not fully capture the metabolic complexity of soil fungal communities. Future research should employ metagenomics or transcriptomics to obtain higher resolution functional profiles. Field-based trials were subject to environmental variability, such as uncontrolled temperature and moisture conditions, and limitations in nutrient bag uniformity, which could influence fungal community dynamics and cultivation outcomes.

## 5. Conclusions

This study analyzed the composition of soil fungal communities at different growth stages of *Morchella sextelat* using high-throughput sequencing technology. The results indicated that the diversity, composition, and relative abundance of fungal communities during the cultivation period of *Morchella sextelat* exhibited significant differences and stage-specific characteristics across various growth stages. At the genus level, the dominant genus was *Morchella* during the LS and LC stages throughout the entire growth cycle of *Morchella sextelat*, whereas *Paecilomyces* was predominant during the LY stages. The most significant changes in fungal community abundance occurred during the LY and LC stages, accompanied by an increase in the abundance of potential pathogenic fungi. At the phylum level, *Ascomycota* consistently dominates during the succession process. Functional predictions of soil fungal microorganisms revealed that saprotrophic fungi predominated, with a gradual increase in the relative abundance of pathotrophic fungi. This study unveiled a dynamic overview of soil fungal communities during the growth process of *Morchella sextelata*, including their characteristics and variation patterns at different growth stages. The findings can provide scientific insights for optimizing artificial cultivation techniques of *Morchella sextelata* and serve as a reference for disease prevention.

## Data Availability

The original contributions presented in this study are included in the article. Further inquiries can be directed to the corresponding authors.
